# Effects of Exogenous Inoculation on Microbial Community Dynamics and Maturation Process in Cattle Manure Composting

**DOI:** 10.3390/microorganisms14030629

**Published:** 2026-03-11

**Authors:** Yufu Hu, Yilin Yuan, Sen Qi, Shuaiqi Feng, Jiamin Yin, Zhuo Xin, Hongyan Zhao, Xin Wang, Zongjun Cui

**Affiliations:** 1 College of Agriculture, Yanbian University, Yanji 133002, China; 2023010604@ybu.edu.cn (Y.H.); onealim@163.com (Y.Y.); 2023050896@ybu.edu.cn (S.Q.); 2025001112@ybu.edu.cn (S.F.); 2023050882@ybu.edu.cn (J.Y.); dks71yy@163.com (Z.X.); 2 College of Agriculture, China Agricultural University, Beijing 100193, China; acuizj@cau.edu.cn

**Keywords:** micro-aerobic composting, lignocellulose-degrading microbial agent, commercial microbial agent, compost maturity, microbial community

## Abstract

Cattle manure composting is an effective strategy for recycling agricultural waste. However, the presence of lignocellulosic materials in cattle manure–maize straw mixtures can limit the degradation efficiency during composting. This study investigated the effects of microbial inoculation on composting performance using three treatments: a lignocellulose-degrading microbial consortium (MC1), a commercial microbial inoculant (BS1), and a non-inoculated control (CK). The results showed that the MC1-treated pile entered the thermophilic phase (>50 °C) earlier than the BS1-treated pile. After 49 days of composting, the lignocellulose degradation rates in the MC1, BS1, and CK treatments were 46.25%, 37.5%, and 29.8%, respectively. Based on compost maturity indicators, including temperature, C/N ratio, pH, and electrical conductivity (EC), the composting period required to reach maturity was shortened by 8 days in the MC1 treatment compared with the BS1 treatment (37 vs. 45 days). Microbial community analysis indicated that MC1 inoculation increased the relative abundance of key microbial groups, particularly Ascomycota and Firmicutes, thereby enhancing lignocellulose degradation and accelerating composting. These findings provide insights into the application of lignocellulose-degrading microbial inoculants for improving cattle manure composting efficiency.

## 1. Introduction

The rapid expansion of livestock farming has created significant environmental and agricultural sustainability challenges regarding animal manure treatment and resource utilisation [1]. Global livestock manure production is nearly 6.3 billion tonnes annually, with China accounting for over one-fifth of this total through its annual output of 1.38 billion tonnes of cattle manure [2,3]. Currently, the disposal of these wastes primarily involves stacking, which can lead to serious water, soil and air pollution [4]. Effectively managing cattle manure has thus become pivotal to advancing China’s livestock sector [5]. Microaerophilic composting represents a vital pathway for resource recovery from cattle manure. This process facilitates the degradation of lignocellulose within manure compost, converting it into humus and nutrients [6]. However, both cattle manure and maize straw contain substantial quantities of recalcitrant organic carbon components, such as the lignocellulosic components hemicellulose, cellulose, and lignin [7]. As the major constituents of lignocellulose, cellulose and hemicellulose degradation presents a significant obstacle to the large-scale industrial application of biological composting. This is attributed to the highly resistant molecular hydrogen bonds among glucose units, resulting in the formation of compounds characterized by high crystallinity, mechanical strength, and chemical stability [8]. Consequently, its degradation constitutes the primary rate-limiting step influencing composting duration [9]. Thus, enhancing the biodegradation of lignocellulose holds significant importance for improving both compost quality and efficiency.

The inoculation of exogenous microorganisms in cattle manure composting enhances the diversity and abundance of lignocellulose-degrading microbes, stimulates microbial activity, accelerates lignocellulose biodegradation, and improves compost maturity and quality [10]. Furthermore, exogenous microbial inoculation reduces odour emissions, effectively suppresses harmful microbial proliferation, and renders the composting process more environmentally sustainable and hygienic [11]. Recently, fungal and bacterial degradation of lignocellulose has garnered increasing attention. Fungi are primary producers of lignocellulases, whereas bacteria typically exhibit faster growth rates and greater thermal tolerance for these enzymes [6]. Multiple studies have screened and identified microorganisms that promote cellulose degradation; applying these specific functional microbes to composting enhances lignocellulose breakdown [12,13,14]. Specific lignocellulose-degrading bacteria can enhance lignocellulose degradation efficiency and compost quality [15]. Yin et al. [16] added the PLC-8 microbial inoculant, resulting in significant enrichment of Proteobacteria and Ascomycota. After 60 days, the degradation rates of cellulose and hemicellulose reached 88.04% and 71.95%, respectively. Wei et al. [17] inoculated with actinomycetes during composting to accelerate lignin and cellulose decomposition. Wang et al. [2] inoculated with *Bacillus*, increasing the degradation rates of cellulose, hemicellulose, and lignin from 17.1–46.4%, 16.9–34.5%, and 30.6–69.4%, respectively. Wang et al. [18] added microbial inoculants (*Bacillus roheii*, *Pseudomonas* spp., and *Aeromonas* spp.) to efficiently degrade lignocellulosic components, generating humic precursors and promoting humic substance formation. In straw composting, inoculation with *Geobacillus stearothermophilus* B5 and *Aspergillus fumigatus* Z5 reduced cellulose content from 43% to approximately 20% and hemicellulose content from 37% to approximately 15%, thereby enhancing composting efficiency and maturity [19]. Zhu et al. [20] inoculated *Gloeophyllum trabeum* into pig manure and wheat straw, reducing the compost maturation period from 39 to 30 days while increasing the degradation rates of hemicellulose, lignin, and cellulose by 49.4%, 109.4%, and 181.1%, respectively. These findings highlight the potential of exogenous microorganisms in accelerating lignocellulose degradation and enhancing compost quality.

Although numerous studies have demonstrated the benefits of exogenous microbial inoculation in enhancing composting efficiency and lignocellulose degradation, most of these studies have focused on single microbial strains or commercially available inoculants [21,22,23]. Comparative analyses between self-developed lignocellulolytic microbial agents and commercial microbial inoculants are still limited. Furthermore, systematic investigations linking microbial community succession to the degradation dynamics of cellulose, hemicellulose, and lignin throughout the composting process remain scarce. Therefore, this study aimed to evaluate the effects of different microbial inoculants on lignocellulose degradation and microbial community dynamics during cattle manure composting. The results are expected to provide insights for optimizing microbial inoculation strategies and improving compost quality and efficiency.

## 2. Materials and Methods

### 2.1. Composting Materials and Experimental Design

The composting experiment was conducted at Yanbian University (Yanji City, China, 43.10° E, 129.18° N), which is located in a temperate semi-humid climate zone. Fresh cattle manure and corn straw were mixed at a mass ratio of 3:1 (*w*/*w*) and thoroughly homogenized to prepare the composting substrate. Three treatments were established: a non-inoculated control (CK) and two inoculated treatments with a lignocellulose-degrading microbial consortium (MC1) or a commercial microbial inoculant (BS1). The lignocellulose-degrading microbial consortium MC1, consisting mainly of members of the phyla Proteobacteria, Firmicutes, Bacteroidetes, Basidiomycota, and Actinobacteria, was provided by the research group of Prof. Zongjun Cui at China Agricultural University [24]. The commercial microbial inoculant BS1 (Haowangnong EM fermentation agent, Zhengzhou, China) contains *Bacillus subtilis* (>1 × 10^10^ CFU g^−1^) and yeast (>3 × 10^9^ CFU g^−1^). For the inoculated treatments, 2 kg of wheat bran was added to each compost pile as a carrier to facilitate microbial activation. Subsequently, 10 mL of MC1 culture (3 × 10^8^ CFU mL^−1^) or BS1 inoculant (containing approximately 5 × 10^8^ CFU mL^−1^ of *Bacillus subtilis* and 1.5 × 10^8^ CFU mL^−1^ of yeast) was applied and thoroughly mixed to ensure uniform distribution throughout the composting materials.

### 2.2. Composting Method and Sampling

**Figure 1:**
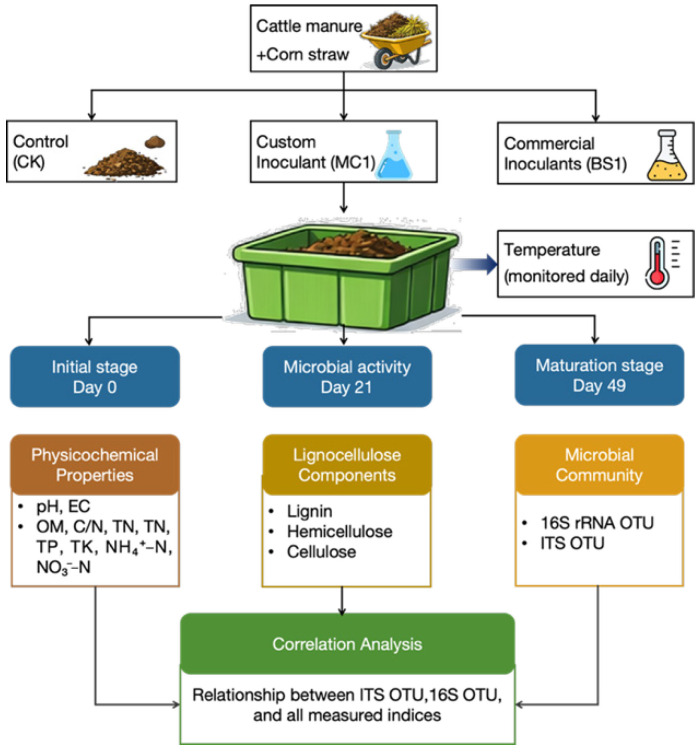
Schematic diagram of the experimental design for cattle manure composting, showing treatments (CK, BS1, MC1), sampling time points (Day 0, 21, 49), and measured parameters.

Each treatment was composted in a separate pile with a volume of 2 m^3^ and a height of approximately 1 m under natural fermentation conditions. When the pile temperature reached 50 °C, manual turning was conducted every 2 days. After the temperature dropped to 35 °C, turning was conducted every 5 days. The composting process lasted 49 days, with Day 0 defined as the start of composting. Samples were collected on Days 0, 21, and 49 at depths of 30, 80, and 100 cm from the pile surface. These sampling points were selected to represent the initial stage (Day 0), the thermophilic stage with the highest microbial activity (Day 21), and the maturation stage (Day 49) of the composting process. The collected samples were thoroughly homogenized, air-dried in the shade, and stored for subsequent analysis [16]. A Schematic diagram of the experimental design, showing treatments, sampling time points, and measured parameters, is shown in Figure 1.

### 2.3. Pile Temperature Measurement

Pile temperature was measured daily at 12:00 throughout the composting period. Temperature sensors were inserted at depths of 20, 50, and 70 cm below the pile surface, and the mean value was recorded. Ambient temperature was measured simultaneously.

### 2.4. Chemical Parameter Analysis

pH measurements were conducted using a pH meter (pH-100A, Lichen Technology Co., Ltd., Shanghai, China). Samples were diluted with distilled water at a ratio of 1:5 (*w*/*v*) before measurement [25]. For the analysis of total potassium (TK) and total phosphorus (TP) components in the soil, a digestion method involving concentrated perchloric acid and sulfuric acid was employed [26]. The digested solution was filtered through a 0.45 μm PES membrane, and the content was quantified using a flame photometer (WGH series, Lichen Technology Co., Ltd., Shanghai, China) and the AA3 analyzer (AutoAnalyzer 3, SEAL Analytical GmbH, Norderstedt, Germany), respectively. The organic matter (OM) content was determined using the potassium dichromate volumetric method combined with the dilution-heat approach [27]. Total nitrogen (TN) was analysed by digesting samples via the Kjeldahl method, followed by nitrogen content analysis using the AA3 analyzer (AutoAnalyzer 3, SEAL Analytical GmbH, Norderstedt, Germany) [28]. Ammonium nitrogen (NH_4_^+^–N) and nitrate nitrogen (NO_3_^−^–N) were determined by extracting samples with 2 mol/L CaCl_2_ solution and analysing nitrogen components using a continuous flow analyser [29]. Cellulose, hemicellulose, and lignin content were measured by the acid washing method according to the standard procedure [30].

### 2.5. Determination of Microbial Dynamics Indicators

Total genomic DNA was extracted from compost samples using the DNA extraction kit (TIANGEN Biotech, Beijing, China) according to the manufacturer’s instructions. PCR amplification, library construction, and Illumina sequencing were performed by a commercial sequencing platform (Biomarker Technologies Co., Ltd., Beijing, China). The V3–V4 region of the bacterial 16S rRNA gene was amplified using primers 338F (5′-ACTCCTACGGGAGGCAGCA-3′) and 806R (5′-GGACTACHVGGGTWTCTAAT-3′) [31]. The fungal ITS region was amplified using primers ITS1F (5′-CTTGGTCATTTAGAGGAAGTAA-3′) and ITS2 (5′-GCTGCGTTCTTCATCGATGC-3′) [32]. Sequencing was performed on an Illumina MiSeq platform using paired-end 300 bp reads (PE300) (Illumina Inc., San Diego, CA, USA). Raw reads were quality-filtered using fastp with default parameters to remove low-quality sequences and adapter contamination. After quality control, the clean reads showed Q20 values above 99% and Q30 values above 96%, indicating high sequencing quality. Raw sequencing data were processed and analyzed using the BMKCloud platform (Biomarker Technologies Co., Beijing, China), where microbial community diversity analyses and ordination analyses were performed using default parameters. The sequencing statistics, taxonomic tables, and alpha-diversity indices for both bacterial 16S rRNA and fungal ITS datasets are provided in Supplementary Tables S1–S6.

### 2.6. Statistical Analysis

Raw data were processed and subjected to analysis of variance using Excel 2016 (Microsoft Co., Redmond, WA, USA) and SPSS 20.0 (IBM Co., Armonk, NY, USA). Structural equation modelling (SEM) was conducted using IBM SPSS Amos 26.0 (IBM Corp., Armonk, NY, USA). Graphical representations were produced using OriginPro 2021 (OriginLab Corporation, Northampton, MA, USA).

## 3. Results

### 3.1. Temperature Changes During Composting

**Figure 2:**
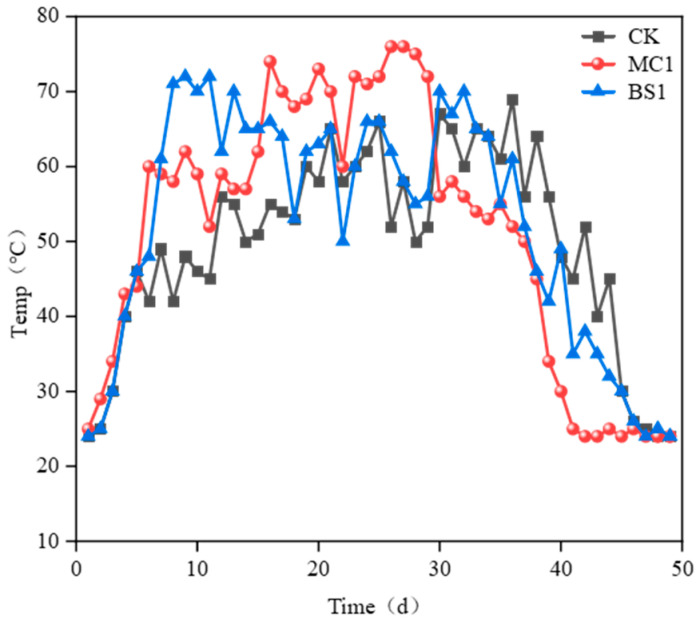
Composting temperature under different treatments during the composting process. MC1 (inoculated with lignocellulolytic microbial agent), BS1 (inoculated with commercial microbial agent), CK (control).

As shown in the compost pile temperature curve (Figure 2), groups inoculated with exogenous microorganisms (MC1, BS1) demonstrated superior performance to the CK group. During the composting heating phase (approximately 0–7 days), temperatures rose rapidly across all treatment groups. The MC1 treatment group entered the high-temperature phase (>50 °C) on day 6, persisting for 31 days, and the BS1 treatment group entered the high-temperature phase on day 7 (7–37 days), lasting 30 days. Both the MC1 and BS1 treatments groups entered the cooling-off period on day 37 (37–49 days), two days earlier than the CK group. The MC1 treatment group reached the high-temperature phase faster than the BS1 treatment group and maintained it for a longer duration.

### 3.2. Physicochemical Properties During Composting

As shown in Table 1, physicochemical indicators exhibited systematic variations across treatment groups throughout the composting process, with significant differences (*p* < 0.05) observed between different stages within the same treatment. pH exhibited a decreasing trend followed by an increase, resulting in a weakly alkaline environment (7.5–8.2) upon completion. Electrical conductivity (EC) initially rose before declining, with all treatments falling below the safety standard of 4 ms/cm at the end of composting [33]. OM content showed an initial increase followed by a decrease. The MC1 treatment achieved the highest OM decomposition rate, reaching 1.44 times that of the BS1 treatment and 2.48 times that of the CK control. The C/N ratio decreased continuously, falling below the maturity standard of 20 in both MC1 and BS1 treatments by the end of composting [34]. TN content showed an upward trend and TP content increased dynamically, while TK content initially increased before decreasing and gradually stabilising. NH_4_^+^-N content initially increased before decreasing, while NO_3_^−^-N content decreased initially before increasing. The experiment demonstrated that adding MC1 significantly promoted OM decomposition, reduced the C/N ratio, and enhanced the retention and transformation of nutrients such as nitrogen and phosphorus.

### 3.3. Lignocellulose Content During Composting

**Figure 3:**
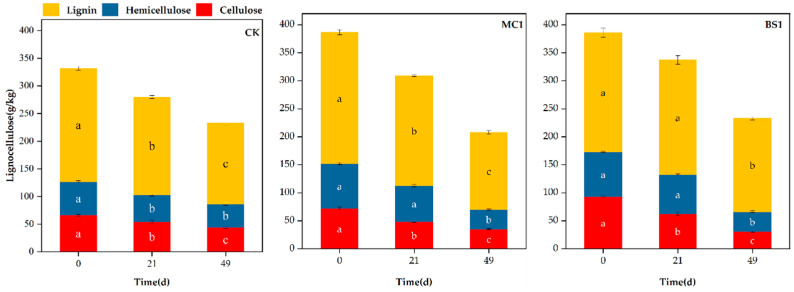
Changes in lignocellulose content under different treatments. MC1 (inoculated with lignocellulose-degrading microbial agent), BS1 (inoculated with commercial microbial agent), CK (control). All data are expressed as mean ± standard error (*n* = 3). Different letters indicated significant differences (*p* < 0.05) according to Duncan’s multiple range test.

The lignocellulose content exhibited a decreasing trend with extended fermentation duration (Figure 3). Upon completion of composting (Day 49), the lignocellulose degradation rates for the MC1, BS1, and CK groups were 46.25%, 37.5%, and 29.8%, respectively. The lignocellulose degradation rate in the MC1 treatment group was 1.23 times and 1.55 times that of the BS1 and CK groups, respectively.

The degradation of cellulose and hemicellulose primarily occurred during the heating and high-temperature phases, indicating that thermophilic microorganisms play a pivotal role in their breakdown. Lignin, being an aromatic compound with a highly complex polymeric structure, is difficult for microorganisms to degrade [19]. By the end (Day 49) of composting, cellulose, hemicellulose, and lignin contents had decreased significantly. Compared to pre-composting levels (Day 0), MC1 treatment reduced these by 51.27%, 56.23%, and 41.32%, respectively; BS1 treatment reduced them by 66.51%, 56.00%, and 17.07%; and CK showed reductions of 33.69%, 29.46%, and 28.65%, respectively. At the end of composting (Day 49), cellulose degradation in the BS1-treated pile was 1.3 and 1.97 times higher than in the MC1-treated and CK piles, respectively; conversely, lignin degradation in the MC1-treated pile was 1.9 and 1.44 times higher than in the BS1-treated and control piles, respectively.

### 3.4. Microbial Community Phylum-Level Composition Analysis

**Figure 4:**
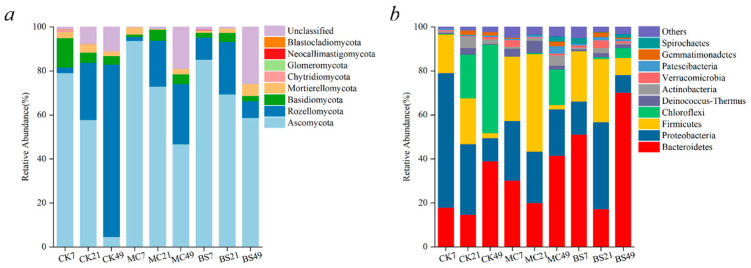
Differences in microbial community composition and dynamics at the phylum level during composting. (**a**) Fungal phyla. MC1 (inoculation with lignocellulolytic microbial agents), BS1 (inoculation with commercial microbial agents), (**b**) Bacterial phyla. Only the ten most abundant phyla are shown; the remaining are grouped as Others. CK (control), with numbers (7, 21, 49) indicating composting time (in days).

Ascomycota constituted the dominant fungal phylum throughout the composting process (Figure 4a). Inoculation with MC1 and BS1 significantly elevated the relative abundance of Ascomycota. The addition of exogenous microorganisms concurrently increased lignocellulose degradation rates, which was accompanied by an increase in Ascomycota abundance, indicating that Ascomycota may play an important role in the observed microbial community dynamics during composting. Moreover, the Firmicutes phylum exhibited high abundance throughout the composting process (Figure 4b). These microorganisms possess thermotolerant and thermophilic properties, thrive in extreme environments, and demonstrate exceptional capabilities in degrading cellulose and hemicellulose [35]. During the composting heating phase and early high-temperature period (0–21 days), degradation microbes within the Ascomycota phylum secreted extracellular oxidase systems such as laccase, manganese peroxidase, and peroxidase. These enzymes are responsible for attacking and breaking down the complex aromatic ring structures of lignin [36]. This breakdown exposes cellulose and hemicellulose, thereby promoting the proliferation of cellulose-degrading bacteria represented by the Firmicutes phylum. At 21 days, the Ascomycota abundance was highest in the MC1 treatment (44.31%), while BS1 and CK treatments recorded 28.6% and 20.84% (Figure 4b), respectively. Throughout the composting process, both Ascomycota and Basidiomycota abundances exhibited a declining trend due to microbial functional shifts, substrate depletion, and temperature dynamics.

### 3.5. Microbial Community Heatmaps

**Figure 5:**
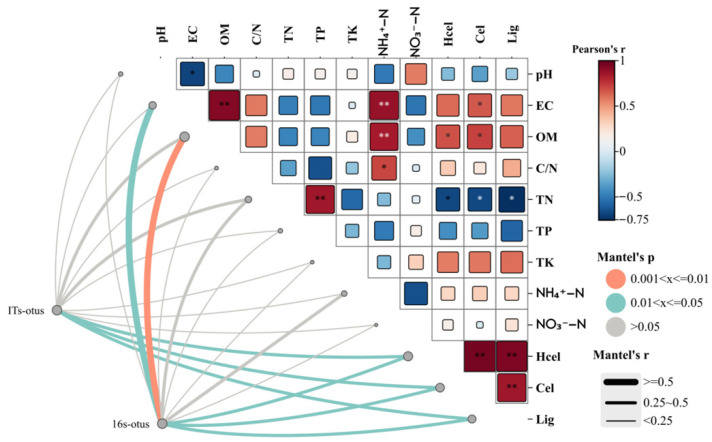
Microbial community correlation heatmap. Rows denote various environmental factors (pH, EC, OM, C/N ratio, TN, TP, TK, NH_4_^+^-N, NO_3_^−^-N, Hcel, Cel, Lig), and columns represent bacterial communities (16S-OTUs) and fungal communities (ITs-OTUs). The colour scale on the right indicates Pearson’s correlation coefficient (Pearson’s r), with red denoting positive correlations and blue representing negative correlations; deeper hues signify stronger associations. The figure also displays Mantel’s correlation coefficient (Mantel’s r) and significance range (Mantel’s p) to assess overall associations with microbial community structure. EC (electrical conductivity), OM (organic matter), TN (total nitrogen), TP (total phosphorus), TK (total potassium), NH_4_^+^-N (ammonium nitrogen), NO_3_^−^-N (nitrate nitrogen), Hcel (hemicellulose), Cel (cellulose), Lig (lignin). * *p* < 0.05, ** *p* < 0.01.

Bacterial communities (indicated as 16S-OTUs) exhibited extremely significant correlations with OM, and significant correlations with lignocellulosic components (indicated as Hcel, Cel, and Lig) and EC (Figure 5). Fungal communities (indicated as ITs-OTUs) showed significant correlations with lignocellulosic components. This indicates that bacterial and fungal community structures undergo corresponding adjustments with variations in lignocellulosic content, likely due to exogenous inoculation promoting bacterial and fungal degradation of lignocellulose, thereby influencing community composition. Furthermore, OM exhibited a highly significant correlation with bacterial communities, underscoring bacteria’s crucial role in accumulating and transforming OM during composting. Cellulose and hemicellulose showed significant positive correlations with OM, potentially linked to OM serving as energy for microbial metabolism. This energy source substantially promotes microbial enrichment, leading to reduced OM content. Consequently, degradation microorganisms extensively break down cellulose and hemicellulose, resulting in corresponding decreases in their concentrations. The lignocellulosic components exhibited significant negative correlations with TN. This result may relate to lignocellulose degradation during composting, wherein elevated TN levels inhibit the synthesis and activity of lignocellulolytic enzymes, thereby suppressing lignocellulose degradation.

### 3.6. Structural Equation Modelling

**Figure 6:**
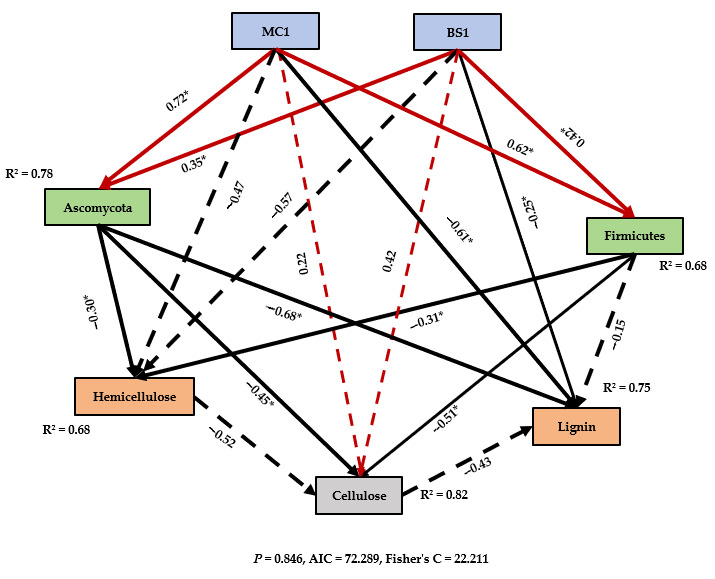
Structural Equation Modelling (SEM) results. This figure illustrates the direct and indirect effects of the lignocellulose-degrading microbial agent (MC1) and commercial inoculum (BS1) on Ascomycota, Firmicutes, and lignocellulose components (cellulose, hemicellulose, and lignin). Numbers beside arrows denote standardised path coefficients, indicating the strength and direction of relationships between variables, with red and black representing positive and negative correlations, respectively (* *p* < 0.05); dashed lines indicate no significant correlation.

According to the SEM analysis in Figure 6, MC1 exerted a significant positive driving effect on the Firmicutes (0.62, *p* < 0.05) and Ascomycota (0.72, *p* < 0.05) phyla. BS1 also demonstrated a significant positive driving effect on the Firmicutes (0.42, *p* < 0.05) and Ascomycota (0.35, *p* < 0.05) phyla, indicating that exogenous microbial addition optimised the microbial community structure within the pile, promoting the enrichment of both Ascomycota and Firmicutes. However, MC1 exhibited higher path coefficients than BS1, suggesting MC1 exerted a more pronounced effect on Firmicutes and Ascomycota than BS1. Ascomycota exhibited significant negative correlations with cellulose (−0.45, *p* < 0.05), hemicellulose (−0.30, *p* < 0.05), and lignin (−0.68, *p* < 0.05). In this study, the significant degradation of lignin was observed in the lignocellulose-degrading bacteria (MC1). Treatment was closely associated with Ascomycota. During the composting process, the relative abundance of Ascomycota exhibited a declining trend, forming a significant negative correlation with the continuous decrease in lignin content. The Firmicutes phylum exhibited significant negative correlations with cellulose (−0.51, *p* < 0.05) and hemicellulose (−0.31, *p* < 0.05), while showing a non-significant negative effect on lignin (−0.15, *p* > 0.05). SEM analysis further revealed the core role of Ascomycota in cellulose degradation. During the composting process, the relative abundance of Ascomycota exhibited an overall trend of initial increase followed by decline, showing significant negative correlations with cellulose and hemicellulose content. Path coefficient analysis indicated that Ascomycota primarily degraded cellulose and hemicellulose. In summary, the lignocellulose-degrading MC1 microbial treatment demonstrated superior lignocellulose degradation performance compared to the commercially available BS1 microbial inoculant.

## 4. Discussion

### 4.1. Changes in Physicochemical Properties During Composting

The findings of this study indicate that the application of exogenous microorganisms significantly influences the physicochemical properties during the composting process and promotes OM transformation, which is closely linked to the dynamic changes in microbial communities within the composting system. Throughout the composting process, the pH values across all treatments fluctuated within the range of 7.5 to 8.2, providing a weakly alkaline environment conducive to microbial growth [37]. The pH values across treatments initially decreased before increasing, a phenomenon linked to organic acid production and ammonification [38]. Organic acid generation inhibited microbial activity, leading to increased H^+^ release and pH decline; conversely, ammonification produced OH^−^, causing pH elevation—a finding consistent with the observations of Wang et al. [2]. The OM content for all three treatments generally exhibited an initial increase followed by a decrease during composting, consistent with the findings of Wei et al. [39]. The transformation of organic carbon in composting primarily involves three stages: degradation, CO_2_ release, and microbial carbon fixation [39,40]. The initial increase in OM content may be attributed to the concentration effect caused by moisture loss and the preferential degradation of easily degradable components [41]. The addition of lignocellulose-degrading bacteria (MC1) demonstrated superior degradation efficacy compared to commercially available microbial inoculants (BS1). The enhanced OM degradation observed in the MC1 treatment may be attributed to the higher lignocellulose-degrading capacity of the inoculated microorganisms, which likely accelerated the breakdown of recalcitrant carbon fractions such as cellulose and hemicellulose [42]. This accelerated degradation may also have contributed to the higher TN concentration observed in the MC1 treatment through increased organic matter mineralization. TN exhibited an overall upward trend for the MC1 and BS1 groups, with organic nitrogen constituting the predominant fraction of TN in compost [43,44,45]. During decomposition, organic nitrogen undergoes mineralisation and decomposition. As inorganic nitrogen converts to ammonia and nitrous oxide, TN content gradually diminishes. Mineralization of organic matter results in a concentration effect. As compost mass decreases during composting, TN concentration typically increases [35]. This trend is consistent with Yang et al. [46].

### 4.2. Changes in Lignocellulose During Composting

Lignocellulose constitutes the major fraction of organic matter in composting substrates such as cattle manure and maize straw and is inherently recalcitrant to microbial degradation [22]. During composting, microbial activity progressively breaks down lignocellulosic components through the secretion of extracellular enzymes, including cellulases, hemicellulases, and lignin-degrading oxidases [47,48]. As composting progressed, lignocellulose content decreased in all treatments, although significant differences in degradation efficiency were observed among treatments. The commercial inoculum BS1 demonstrated robust cellulose degradation capacity. This capability is likely associated with the presence of cellulolytic bacteria such as *Bacillus subtilis*, which are known to secrete cellulases that hydrolyze cellulose into soluble sugars [49,50,51]. However, the relatively low lignin degradation observed in the BS1 treatment suggests that the microbial consortium lacked efficient ligninolytic microorganisms, which limited the overall lignocellulose degradation efficiency. In contrast, the MC1 treatment showed enhanced lignin degradation, indicating the potential presence of microorganisms capable of producing ligninolytic enzymes. Members of *Ascomycota*, which were enriched in the MC1 treatment, are known producers of enzymes such as laccase and peroxidase that catalyze oxidative lignin depolymerization [52]. The breakdown of lignin is particularly important because lignin forms a complex protective matrix that surrounds cellulose and hemicellulose, restricting microbial access to these polysaccharides [53,54,55]. Therefore, efficient lignin depolymerization in the MC1 treatment likely disrupted this structural barrier, increasing the accessibility of cellulose and hemicellulose to microbial enzymes and thereby promoting overall lignocellulose transformation [56,57]. This finding suggests that lignin degradation represents a key limiting step in lignocellulose conversion during composting, and microbial consortia with strong ligninolytic activity may substantially enhance composting efficiency.

### 4.3. Changes in Microbial Community During Composting

Composting represents a dynamic microbial-driven ecological succession process in which shifts in microbial communities directly influence organic matter transformation and compost maturation [58,59]. Previous studies have shown that exogenous microbial inoculation can reshape microbial community structure by enriching specific functional groups and thereby improving degradation pathways [60,61]. The lignocellulose-degrading inoculum (MC1) increased the relative abundance of Proteobacteria and Firmicutes during the warming and thermophilic phases (Figure 4b), whereas the commercial inoculum (BS1) exhibited higher Proteobacteria abundance mainly during the thermophilic phase. Proteobacteria are commonly abundant in organic-rich environments and are frequently associated with organic matter transformation, including the degradation of cellulose and hemicellulose and the release of mineral nutrients and intermediate metabolites that support microbial growth [62,63]. Numerous members of Firmicutes are facultative or obligate anaerobic cellulolytic bacteria capable of degrading cellulose and hemicellulose under the micro-anaerobic conditions commonly found in composting systems. Their increased relative abundance during the warming and thermophilic phases was associated with the rapid depletion of cellulose-based components [64]. Ascomycota represented the dominant fungal group throughout the composting process (Figure 4a). SEM analysis indicated that MC1 exerted a stronger positive effect on Ascomycota (path coefficient = 0.72, *p* < 0.05) compared with BS1 (path coefficient = 0.35, *p* < 0.05) (Figure 6). Members of Ascomycota are widely recognized as important contributors to lignin degradation in composting systems [65]. These fungi are capable of degrading lignin through the secretion of ligninolytic enzymes such as laccase and manganese peroxidase and exhibited a significant negative correlation with lignin content (path coefficient = −0.68, *p* < 0.05) in this study. Efficient lignin degradation may enhance the accessibility of cellulose and hemicellulose for subsequent degradation by other microorganisms, including members of Actinomycetota [36]. Overall, the addition of MC1 promoted the enrichment of key functional microbial groups associated with lignocellulose degradation, particularly lignin-degrading microorganisms, thereby facilitating overall lignocellulose transformation and accelerating the compost maturation process.

## 5. Conclusions

This study demonstrated that inoculation with lignocellulose-degrading microorganisms (MC1) and a commercial microbial inoculant (BS1) improved the composting performance of cattle manure and corn stover. Among the treatments, MC1 showed superior effectiveness in promoting lignocellulose degradation and accelerating compost maturation. The MC1 treatment optimised the microbial community structure, particularly by enriching Ascomycota and Firmicutes, which are closely associated with lignocellulose decomposition and the production of ligninolytic enzymes. Enhanced lignin degradation in the MC1 treatment likely facilitated the subsequent breakdown of cellulose and hemicellulose, thereby improving the overall efficiency of lignocellulose transformation during composting. Physicochemical indicators, including temperature, C/N ratio, pH, and EC, suggested that MC1-amended compost reached maturity earlier than the BS1 and control treatments, indicating an accelerated composting process. Overall, these findings highlight the importance of microbial consortia with strong ligninolytic potential for overcoming the recalcitrance of lignocellulosic materials in composting systems. The results provide a theoretical basis and practical guidance for developing effective microbial inoculants to enhance the efficiency and sustainability of agricultural waste composting.

## Figures and Tables

**Table 1:** Physicochemical parameters during the composting process.

Time	Processing	pH	EC (ms/cm)	OM (g/kg)	C/N	TN (g/kg)	TP (g/kg)	TK (g/kg)	NH_4_^+^-N (mg/kg)	NO_3_^−^-N (mg/kg)
0 d	CK	7.93 ± 0.05 a	5.07 ± 0.36 a	872.46 ± 8.42 a	32.42	16.82 ± 0.15 c	7.87 ± 0.22	39.2 ± 0.03 a	40.13 ± 0.51 b	275.15 ± 3.74 a
	MC1	7.93 ± 0.05 a	5.07 ± 0.36 a	872.46 ± 8.42 b	32.42	16.82 ± 0.15 c	7.87 ± 0.22 b	39.2 ± 0.03 a	40.13 ± 0.51 b	275.15 ± 3.74 a
	BS1	7.93 ± 0.05 b	5.07 ± 0.36 b	872.46 ± 8.42 b	32.42	16.82 ± 0.15 c	7.87 ± 0.22 b	39.2 ± 0.03 a	40.13 ± 0.51 b	275.15 ± 3.74 b
21 d	CK	7.72 ± 0.02 b	5.76 ± 0.25 a	889.52 ± 10.65 a	22.78	24.41 ± 0.37 a	7.91 ± 0.24	20.2 ± 0.27 c	55.5 ± 1.45 a	115.06 ± 6.32 bc
	MC1	7.61 ± 0.01 b	5.35 ± 0.14 a	912.13 ± 5.56 a	21.47	26.55 ± 0.23 b	8.72 ± 0.29 b	38.73 ± 0.25 b	48.7 ± 0.66 a	163.05 ± 3.19 c
	BS1	7.52 ± 0.03 c	6.26 ± 0.43 a	900.31 ± 5.56 a	21.78	25.84 ± 0.27 b	8.72 ± 0.29 ab	30.6 ± 0.21 b	49.78 ± 2.73 a	142.94 ± 4.55 c
49 d	CK	7.92 ± 0.03 a	3.95 ± 0.06 b	809.65 ± 6.35 b	24.38	20.76 ± 0.34 c	8.21 ± 0.21	31.53 ± 0.16 b	38.73 ± 0.26 b	146.96 ± 5.94 b
	MC1	8.01 ± 0.02 a	3.47 ± 0.09 b	714.42 ± 1.72 c	13.6	32.82 ± 0.18 a	10.01 ± 0.27 a	28.2 ± 0.2 c	30.34 ± 0.18 c	208.35 ± 4.54 bc
	BS1	8.15 ± 0.02 a	3.68 ± 0.31 c	763.17 ± 0.98 c	15.37	31.04 ± 0.2 a	9.13 ± 0.32 a	32.2 ± 0.21 b	31.89 ± 0.26 c	523.175 ± 5.87 a

Note: MC1 (inoculated with lignocellulolytic microbial agents), BS1 (inoculated with commercial microbial agent), CK (control), EC (electrical conductivity), OM (organic matter), TN (total nitrogen), TP (total phosphorus), TK (total potassium), NH_4_^+^-N (ammonium nitrogen), NO_3_^−^-N (nitrate nitrogen). Values in the table represent the mean of three replicates per sample. Different letters denote significant differences between treatments within the same column (*p* < 0.05).

## Data Availability

The original data presented in the study are openly available in Zenodo at https://doi.org/10.5281/zenodo.18465542.

## Supplementary Materials

The following supporting information can be downloaded at: https://www.mdpi.com/article/10.3390/microorganisms14030629/s1, Table S1: 16S rRNA sequencing CCS reads statistics per sample; Table S2: 16S rRNA sample-level taxonomic (Kingdom–Species) OTU counts; Table S3: 16S rRNA alpha-diversity indices; Table S4: Fungal ITS sequencing CCS reads statistics per sample; Table S5: Fungal ITS sample-level taxonomic (Kingdom–Species) OTU counts; Table S6: Fungal ITS alpha-diversity indices.
